# The Effect of Spaceflight on Growth of *Ulocladium chartarum* Colonies on the International Space Station

**DOI:** 10.1371/journal.pone.0062130

**Published:** 2013-04-24

**Authors:** Ioana Gomoiu, Elias Chatzitheodoridis, Sonia Vadrucci, Isabelle Walther

**Affiliations:** 1 Institute of Biology Bucharest, Romanian Academy of Science, Bucharest, Romania; 2 School of Mining and Metallurgical Engineering, National Technical University of Athens, Athens, Greece; 3 Space Biology Group, ETH Zürich, Zürich, Switzerland; California Department of Public Health, United States of America

## Abstract

The objectives of this 14 days experiment were to investigate the effect of spaceflight on the growth of *Ulocladium chartarum*, to study the viability of the aerial and submerged mycelium and to put in evidence changes at the cellular level. *U. chartarum* was chosen for the spaceflight experiment because it is well known to be involved in biodeterioration of organic and inorganic substrates covered with organic deposits and expected to be a possible contaminant in Spaceships. Colonies grown on the International Space Station (ISS) and on Earth were analysed post-flight. This study clearly indicates that *U. chartarum* is able to grow under spaceflight conditions developing, as a response, a complex colony morphotype never mentioned previously. We observed that spaceflight reduced the rate of growth of aerial mycelium, but stimulated the growth of submerged mycelium and of new microcolonies. In Spaceships and Space Stations *U. chartarum* and other fungal species could find a favourable environment to grow invasively unnoticed in the depth of surfaces containing very small amount of substrate, posing a risk factor for biodegradation of structural components, as well as a direct threat for crew health. The colony growth cycle of *U. chartarum* provides a useful eukaryotic system for the study of fungal growth under spaceflight conditions.

## Introduction

Although organisms can survive and reproduce in space, spaceflights expose all organisms to several major stress conditions, such as space radiation, microgravity and other low shear environment. These factors affect their survival rate and physiology. Microorganisms are present ubiquitously and on the Space Station. They can be found as contaminants coming from the Earth, as part of experiments and as normal microbiota from crew members. On the International Space Station, *Staphylococcus* sp was identified as the most dominant airborne bacterial genus, *Aspergillus* sp, *Penicillium* sp and *Cladosporium* sp as the most dominant genera in the fungal population [1], [2], [3]. In the Japanese experimental module KIBO on the ISS, *Alternaria* sp and *Malassezia* sp were the dominant species before launch and in space [4]. Other opportunistic pathogens and species involved in biodeterioration of structural materials or biodegradation were identified in food and waste storage, and recycling systems [5], [6]. All these studies show that the environment on board the ISS allows the growth of fungi. Better knowledge on the influence of spaceflight on microorganisms’ development will help in taking the correct countermeasure to increase the chance of having good living conditions for the crew during long duration flights. Studies on the behaviour of microorganism to space conditions have been performed on bacteria [7], [8], [9], [10], [11] and yeast [12], [13], [14]. Almost no information is available on how fungi are affected and changed due to growth under space conditions. To evaluate the risks of causing infections or allergies by fungi on board spacecraft, it is necessary to understand and predict the growth in the new environment and how their characteristics change in space.

Morphological and phenotypical changes were found in the fungus *Pleurotus ostreatus* under simulated microgravity conditions [15]. Yamazaki et al [16] on the other side, did not find any differences in morphology, asexual reproductive capability, or susceptibility to antifungal agents in strains of the pathogenic fungi *Aspergillus niger* and *Candida albicans* using a three dimensional clinostat to simulate microgravity. Altenburg et al [17] found that *Candida albicans* responds to simulated microgravity by an increase in filamentous forms and changes in expression of two genes associated with the yeast-hyphal transition. In the same experimental conditions it was found that these morphological and molecular changes are in conjunction with other observation such as increased pathogenicity [18]. Post-landing analysis of soybean roots contaminated with *Phytophtora soja* showed no detectable morphological differences in the fungal strain between spaceflight and gravity conditions [19].

Fungi are sensitive to changes in direction of the gravity vector (gravitropism) [20]. Fruiting bodies of *Flammulina velutipes* grown in microgravity exhibited random orientation but flat and helically twisted stipes were noticed [21]. The differentially expressed genes responding to gravitational change are involved in potential cellular mechanisms during fruiting body developing of *Pleurotus ostreatus* [15].

Normally, submerged mycelium grows simultaneously with aerial mycelium assuring the growth of the colony. A highly polarized growth promotes invasion of the substrate or the host in case of pathogenic species [22]. An increase in tip yielding can hinder the turgor pressure to become bigger and lead to an invasive hyphal growth [23]. Depletion of nutrients, presence of toxins or microgravity can induce invasive growth as an adaptive response either to forage the substrate or to avoid chemical and physical effect. As an example, diploid cells of *Saccharomyces cerevisiae* change their growth from budding to pseudohyphae when starved for nitrogen [24], [25]. In a space experiment, van Mulders et al showed that certain strains grew invasively but with reduced growth in the centre of the colony [13].

The sensitivity of fungi to their environment, the ease to cultivate them, and their well-known metabolism and molecular biology make these organisms good models for space experiments. The species *Ulocladium chartarum* has been chosen for this space flight experiment because it is well known to be involved in biodeterioration of organic and inorganic substrates covered with organic deposits and expected to be a possible contaminant in space stations. *Ulocladium botrytis* for example was isolated by Novikova et al [1] next to *Aspergillus niger* and *Cladosporium herbarum* in samples taken from structural elements and internal surfaces of Soyuz taxi-flights and ISS between 1998–2005**.** Another reason to choose *U. chartarum* is the fact that it is also supposed to be resistant to space radiation due to its melanin content in hyphae and spores [26], [27], [28].

The objective of the 14 days spaceflight experiment was: first to investigate the effect of spaceflight on the colonies of different age and hyphae growth of *U. chartarum*, second to study the viability of the aerial and submerged mycelium and third to put in evidence changes at the cellular level.

## Materials and Methods

### Biological Material

*Ulocladium chartarum CM*-1 has been isolated from mural paintings, mainly from the vegetal residues contained on the intonaco layer (Romania); it is deposited as IBBCC 41 in the Microbial Culture Collection of the Institute of Biology, Bucharest, Romania and is available to the scientific community. Decimal dilutions were used and inoculation of each dilution was performed on potato-dextrose-agar. The identification of *U. chartarum* has been made according to their macroscopical colonial but also by their microscopic hyphal and spore characteristics [29]. For the here presented study the strain was cultivated on YGC substrate medium containing 5 mg/ml yeast extract, 20 mg/ml glucose, 0.1 mg/ml chloramphenicol and 20 mg/ml agar.

### Spaceflight Experimental Setup

Living cultures of *U. chartarum* were prepared in polycarbonate culture plates of 60 mm diameter hermetically closed with a Teflon gasket. Substrate (YGC) was inoculated with 10–50 spores at different times before launch (5 days, 3 days, 1 day). The amount of air versus total volume of the culture plate was about 60%.

**Figure 1:**
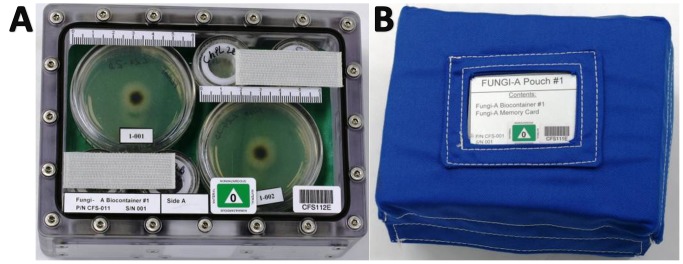
Spaceflight experimental setup. (A) Biocontainer containing fungal cultures and spores. (B) Pouch used for up- and download in Space Shuttle.

Culture plates with living cultures were sealed and assembled into a polycarbonate biocontainer (BC, PedeoTechniek, Belgium). Four large and eight small culture plates (2 of 60 mm and 4 of 30 mm on each side; Figure 1A) were placed into one biocontainer and sealed. BC#1 contained 3 days old, BC#2 1 day old and BC#3, 5 days old colonies. Each of the three biocontainers was equipped with a small programmable miniature-sized temperature data logger (SmartButton from ACR Systems Inc.) as well as passive radiation data loggers (SCK CEN) to monitor ionizing radiation exposure over the space mission. For protection during up- and download, every biocontainer was placed in a pouch of protective foam and NOMEX fabric (Figure 1B).

**Figure 2:**
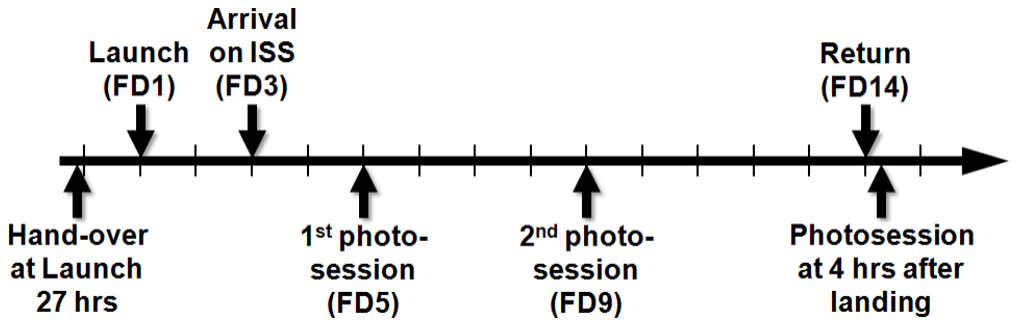
Mission overview.

During the trip to and back from the International Space Station (Shuttle STS-133), as well as on-board ISS the samples were exposed to a temperature of 21.5+/−2°C. Upon arrival to the ISS the biocontainers were transferred to the European Experiment Module “Columbus” where pictures were taken by the Crew on FD (Flight Day) 5 and 9 using a Nikon D2X camera with a 60 mm lens to evaluate the rate of growth. Exposure to light occurred only during the photo sessions. The biocontainers returned un-opened to Earth after 14 days of exposure to spaceflight conditions. Pictures were taken on ground 4 h after return (Figure 2). The total absorbed dose of ionizing radiation recorded during the flight was about 150 *µ*Gy per day (background corrected). An identical set of biocontainers (ground control) as well as samples not integrated into biocontainers (laboratory control), were prepared and kept on Earth as ground control. Pictures of the ground control were taken in parallel to the Crew.

### Colonies’ Morphology

In order to analyse the colonies at different stages of development, culture plates were inoculated at 5 days (BC#3), 3 days (BC#1) and 1 day (BC#2) before launch. The ground controls and flight culture plates were integrated inside biocontainers as indicated before; the laboratory control culture plates were kept inside an incubator. The colonies' morphology was observed and compared among the culture plates after 5 to 18 days of growth.

### Colony Growth

The growth of the colonies was evaluated using images taken by the crew on ISS and by the authors on the ground and after return. The area and the diameter of each colony were measured with the “Image J” software [30]. Measurements were converted into the mm scale using a scale bar attached on the culture plates inside the biocontainers. Average culture diameters and area measurements were acquired from all four culture plates, standard deviation was also calculated. The division of the average colonies’ diameter by the number of days between the photo-sessions gave an estimation of the growth over time. Differences were determined by t-test, p<0.05 was considered significant.

### Microscopy Studies

Microscopy studies for living cultures were performed 3 and 4 days after return from ISS. Both, microscopy imaging and real-time video acquisition were performed only on untreated samples, which were cut from the colonies from flight, ground and laboratory control. For the real-time observations, a trinocular Leica DMLM microscope with highest magnification of ×1000 was used. A set of Leica N PLAN, infinity-corrected objective lenses were also used for viewing at different magnifications, such as at ×5, ×10, ×20 and ×100, through lenses with numerical apertures of 0.12, 0.40, 0.75 and 0.90 respectively.

### Viability Analysis

Viability tests of colonies from each biocontainer were performed 3 and 4 days after return from ISS, immediately after each biocontainer was opened.

Hyphae viability was estimated by scraping aerial mycelium from 5 mm^2^, then inoculated on YGC substrate and incubated for 5 days in a temperature range between 23.5 and 24.5°C. Samples were taken from the edge and centre of the colonies. Viability of submerged mycelium was estimated by cutting an area of 5 mm^2^ near to the margin of the culture plate, not covered by aerial mycelium, then inoculated on YGC substrate treated as above. Samples are considered viable when inoculated hyphae are able to grow, branch and make colonies.

Conidial viability was assessed by scraping spores from an area of 5 mm^2^ aerial mycelium followed by 100-fold dilution and inoculation of decimal dilutions onto 10 cm Petri dish with YGC. After 10 days of incubation, Colony Forming Units (CFU) was counted. Because no new colonies developed, all inoculated culture plates were analysed microscopically to find if hyphae can branch and spores can germinate.

### Hyphal Growth Measurement Tools and Statistics

For the detailed study of hyphal growth and their geometry we have developed specialized software to digitize hyphae and perform statistics in a quantitative rather than a descriptive way. The software is capable of measuring the length of the hyphae and to calculate their degree of curvature. Digitization is performed on optical images acquired with a transmitted light microscope. The software handles images in calibrated scale for the different magnification lenses of the microscope. Each hypha is digitized with a series of line sectors of a certain length (10 µm was used in this work). The total angle is calculated as the sum of the angles defined between each pair of consequent line sectors. The curvature is the total angle divided by the total hyphal length, expressed in units of *degrees/µm*. A number of images were acquired from the margins of the cultures, where growth actually takes place, using the microscope with the smallest magnification ×5. Statistics of the depth distribution of hyphae (depth profiles) is also possible adjusting the focus depths. Digitization of the hyphae is then made in the three-dimensions, represented by three-dimensional vectors, and lengths and angles can be calculated in a similar way as in the two-dimensions. A large number of images were acquired from slices of substrate with the colony, either as general views (magnification ×5) around the edges of the colony, or as depth profiles (stacks of images focusing at consequent depths, starting from the surface). Length and curvature measurements are graphically presented in the form of histograms, both for flight and ground experiments, for each of the three biocontainers. Fitting curves of histograms are moving averages.

Hyphal growth measurements and statistics could be done only for colonies grown in space and the ground control 3 and 4 days after return. In the laboratory control, the maximal growth (up to the rim of the culture plate) was already reached after 5 days of incubation.

## Results

### Colonies’ Morphology, Growth and Viability

**Figure 3:**
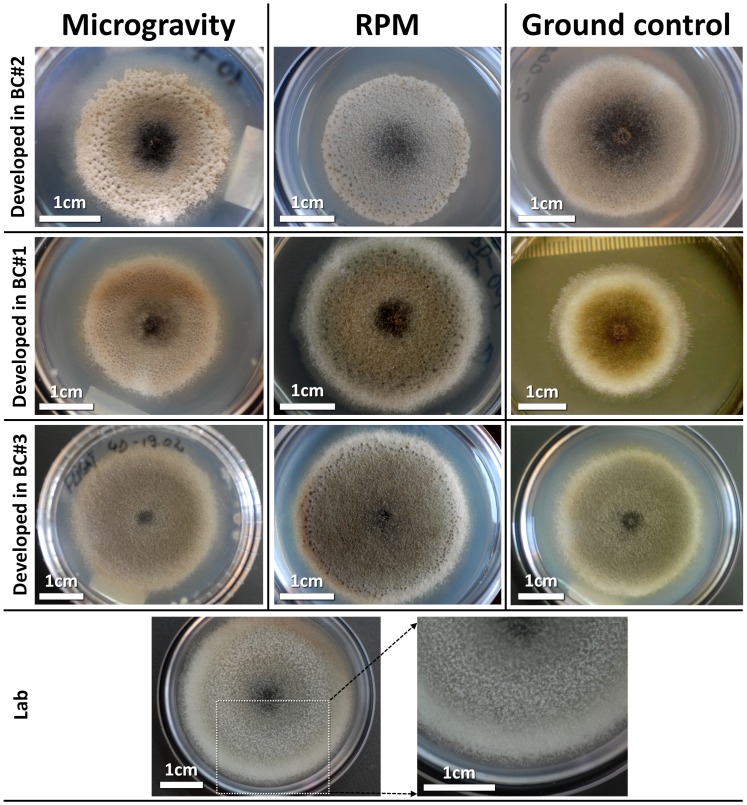
Aspect of *U. chartarum* colonies under different growth conditions.

**Figure 4:**
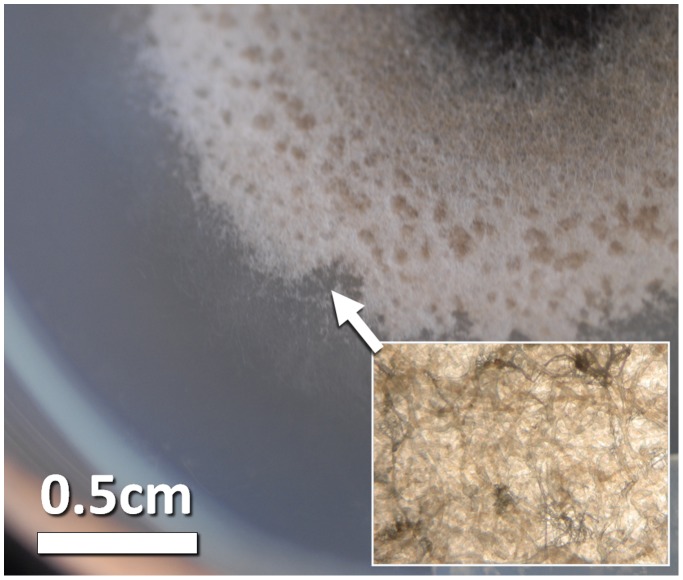
Pitted areas found on the surface of the colony.

The aerial mycelium was observed among the different BC and it showed differences in texture and colour, exudates size and colour, and colony surface and margin; the extension of the submerged mycelium outside the colony margin was also depending on the growth conditions (Table 1). Figure 3 shows clearly that the longer the colony could grow before launch the less pitted areas (Figure 4) and exudate drops were produced (BC#3: 5 days versus BC#2: 1 day). The irregular shape of the colony margin and the presence of pitted areas correlate with the amount of exudates, which keeps secondary metabolites, acting as toxins, near the hyphae. All these observations clearly suggest that the growth conditions have an influence on the morphology of the colonies.

**Table 1:** Comparison of morphology.

	Laboratory control	Ground control in BC		Flight in BC
**aerial mycelium**				
	dense and high		dense and short	less dense and very short
***colour***	olive, black in the centre		green to olive, black in the centre andlight cream towards the margin	brownish to green, black in the centre and light cream towards the margin
***texture***	cotton like		slightly velvety and pitted	velvety, pitted
***margin***	regular		slightly irregular	irregular
***diameter***	very large		medium	large
***exudate drop***	small and few		small and few	large and numerous
***exudate colour***	colourless		colourless	light yellow
**submerged mycelium**				
	not extending outside margin of colony		extending outside margin	extending outside margin

**Figure 5:**
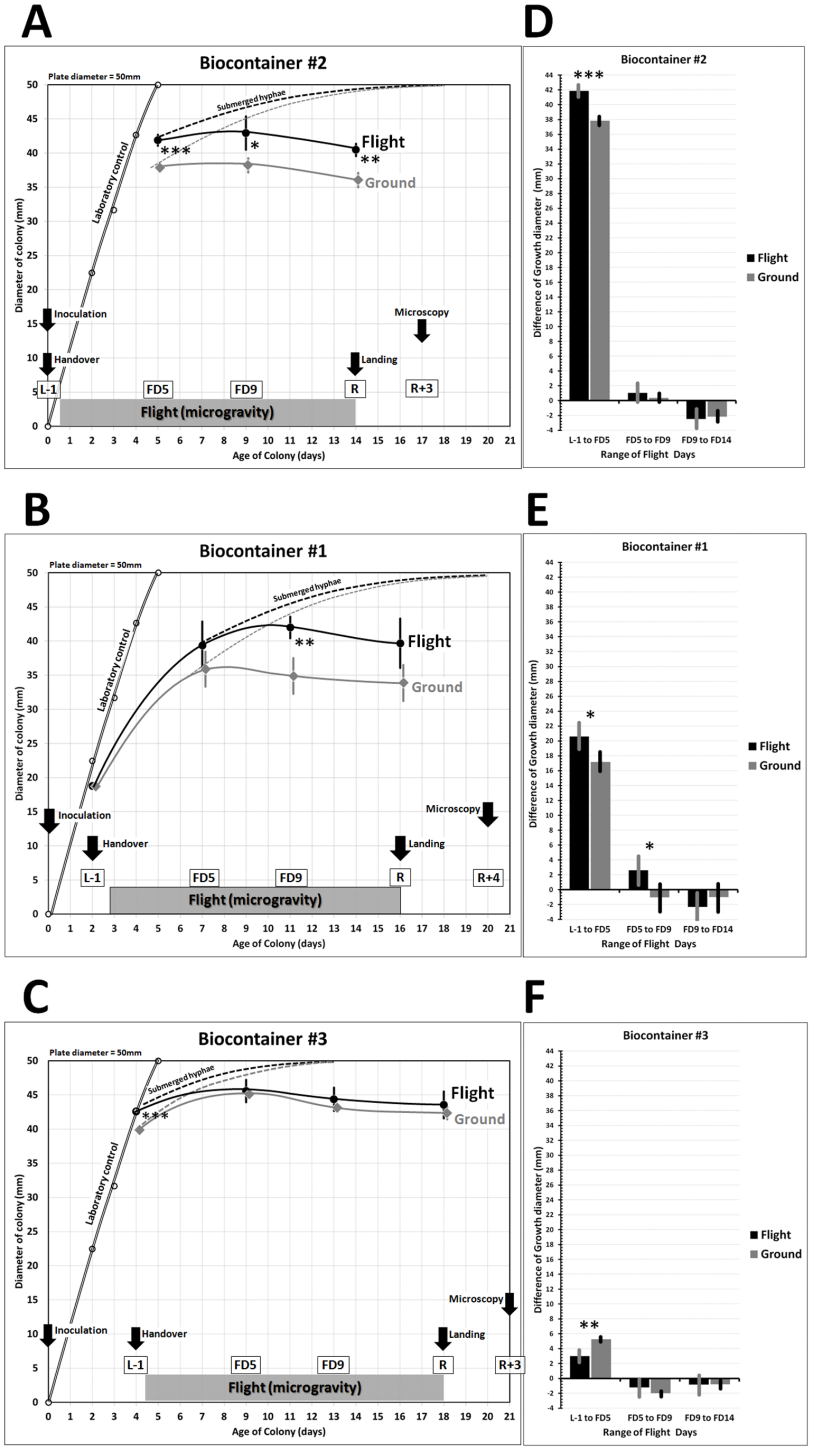
Growth of *U. chartarum* colonies in BC#2, BC#1 and BC#3 (flight, ground) and in laboratory conditions. (A,B,C) Growth curves; (D,E,F) Growth rate. Arrows show the different events, while in boxes “L” means “*Launch*”, “FD” means “*Flight Day*” and “R” means “*Return*”. Solid lines: aerial mycelium diameter; dashed lines: submerged mycelium diameter. Asterisks indicate statistically significant differences between ground and flight samples (*p<0.05; **p<0.01; ***p<0.001); error bars correspond to the standard deviation.

The survival of hyphae, their ability to develop aerial and submerged mycelium as a consequence of branching, and finally their apical growth was evaluated by measuring the diameters of the colonies from pictures taken both on ISS and at the Institute of Biology (Bucharest, Romania). It was found that the growth rate of aerial and submerged mycelium were similar, therefore the overall colony’s growth rate was evaluated using the diameter of the thick aerial mycelium only. As visible in Figure 5 (A, B, C), the diameter of the colonies was always larger in the flight samples than in the ground control, but smaller than in the laboratory control (optimal conditions). Interestingly, the diameter difference between ground control and flight samples seems to be related with the time the colonies were growing before launch. In fact, the longer the colonies developed on ground (BC#3) the smaller the difference. Not only the diameter of the colonies was larger but also the growth rate was mostly higher in flight than on ground (Figure 5 D, E, F). Only colonies in BC#3 showed a lower growth rate of the aerial mycelium in flight than on ground, though the submerged mycelium growth rate in this BC was high. Comparing the BC among each other, it is interesting to see that the highest growth rate was obtained in BC#2 during the 5 first days in space. These colonies have covered a larger surface of the substrate, compared to those from BC#1, which were two days older. Then, between FD5-FD9, the growth rate became very low, even lower than the one of BC#1.

**Figure 6:**
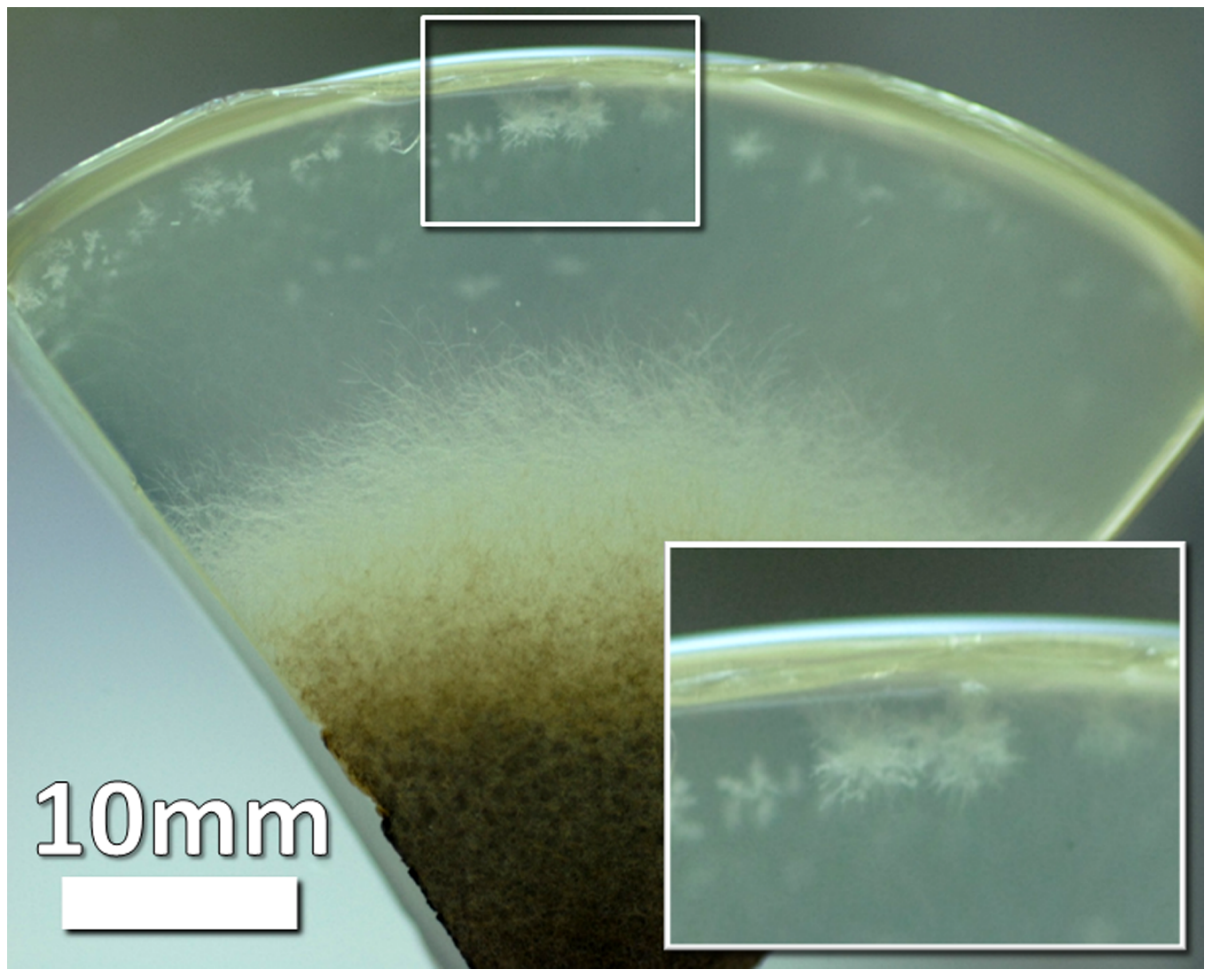
Colony slice showing new colonies grown from the submerged mycelium under spaceflight conditions (box magnified in inset).

Generally, while in the laboratory control, under optimal conditions, full growth was found after five days of incubation, in flight and in the ground control the growth rate was decreasing over time until the aerial growth totally stopped. Growth curves in BC#2, 1 and 3 (Figure 5 A, B, C) showed that the aerial growth stopped between FD4-FD8, with a dependency on the inoculation day. A further growth of the colonies was observed at the level of the submerged mycelium, which gave rise to new colonies initially growing inside the nutrient layer (Figure 6). The formation of such microcolonies was not observed in the ground colonies of the same age. It seems that spaceflight conditions stimulate the growth of submerged mycelium.

All viability tests performed on aerial mycelium and spores of all culture plates (flight and ground) revealed that hyphae from margins and sporulated aerial mycelium were not viable. Microscopic examination of spores showed that about 10% were able to germinate but did not grow further to long and branched hyphae and, in consequence, were not able to form a colony. Submerged mycelium however was always alive and developed normal colonies. Viability tests performed on the laboratory control on the aerial mycelium and spores showed that they are viable.

### Hyphal Growth and Curvature

**Figure 7:**
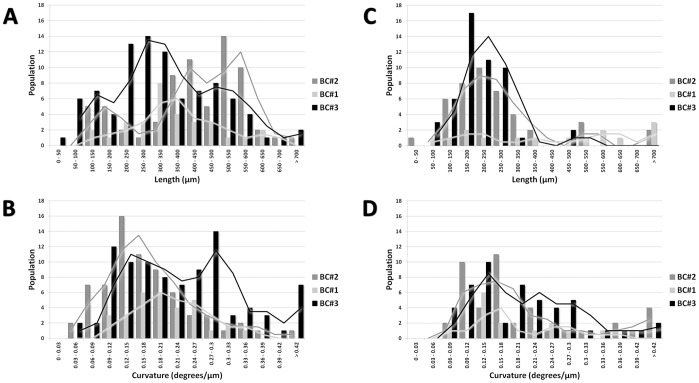
Distribution of hyphal lengths and curvature. Comparison of all three biocontainers both for Flight (A, B) and Ground samples (C, D). Lines are the moving average of the data.

The length of the young hyphae growing at the margin of the colony was measured from the branching point to the tip. The maximal length observed between the different growth conditions was not statistically significant (data not shown), but we observed an interesting difference in the length distribution between ground and flight (Figure 7A, C). The hyphae in the ground BCs were homogeneous and showed an average length of about 200 µm, determined from the fitting curve, where in the flight BCs hyphae were longer and showed a broader distribution. Especially interesting is the flight BC#2, with the colonies grown shortest before flight, i.e.1d; this sample showed the longest hyphae (about 500 µm) with the broadest distribution. It can be deduced, that spaceflight conditions positively influence the growth of the hyphae independently of their age.

**Figure 8:**
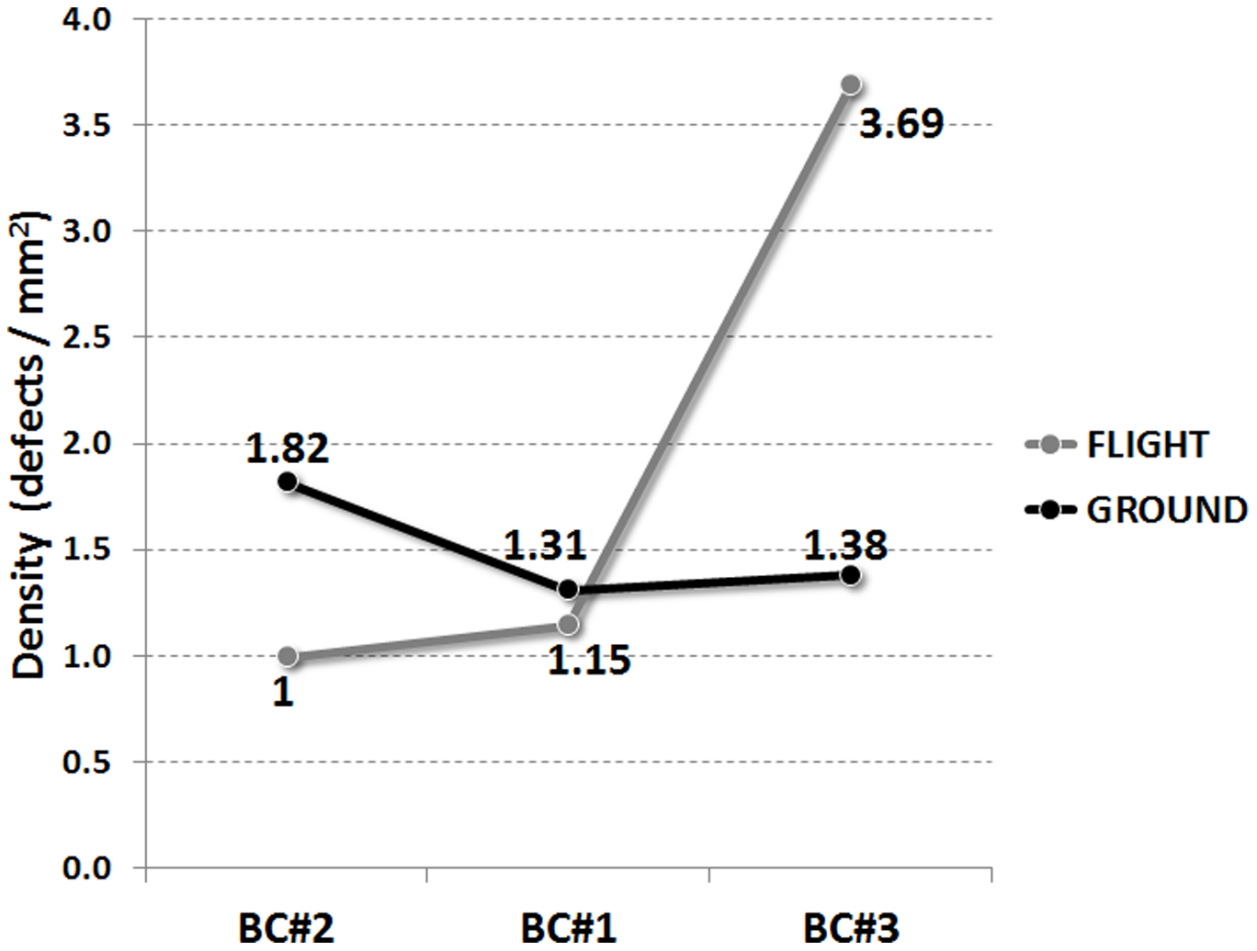
Density of defects (resulting from all hyphae).

The curvature of the hyphae after branching was also investigated (Figure 7B, D). The mean value of the main population is determined from the fitting curve to be about 0.15 degrees/µm for all conditions. In the case of BC#3 (oldest colonies) a second population was observed at almost the double value 0.27 degrees/µm. In flight there was even a third peak of highly curved hyphae (<0.42 degrees/µm – multiple tip folding). Highly curved hyphae are easily damaged and are more prone to defects. This can explain the high density of defects observed in this sample (Figure 8).

**Figure 9:**
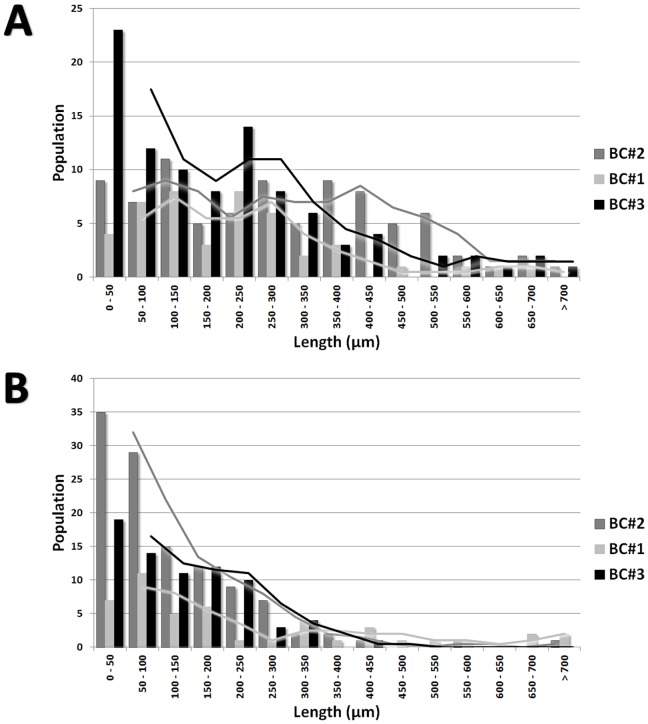
Distribution of branch lengths. (A) Comparison of all three biocontainers both for Flight and (B) Ground samples. Lines are the moving average of the data.

Branches close to the edge of the colony represent young hyphae, which are expected to be good “sensors” of the spaceflight effects, since growth of aerial mycelium had stopped after a certain time under flight conditions. Topologically, branches can grow equally to their parent hyphae from the point they branched, or two branches can evolve from a hyphae tip and grow simultaneously. As for the hyphae distribution, the length distribution of the branches was also much broader in flight than on ground (Figure 9A, B). Interestingly, in flight, the branch length in BC#2 was uniformly distributed up to 400 µm, whereas in BC#3 (longest growth before flight, i.e. 5d) the distribution is similar to the ground sample; and in BC#1 an intermediate behaviour is detected.

**Figure 10:**
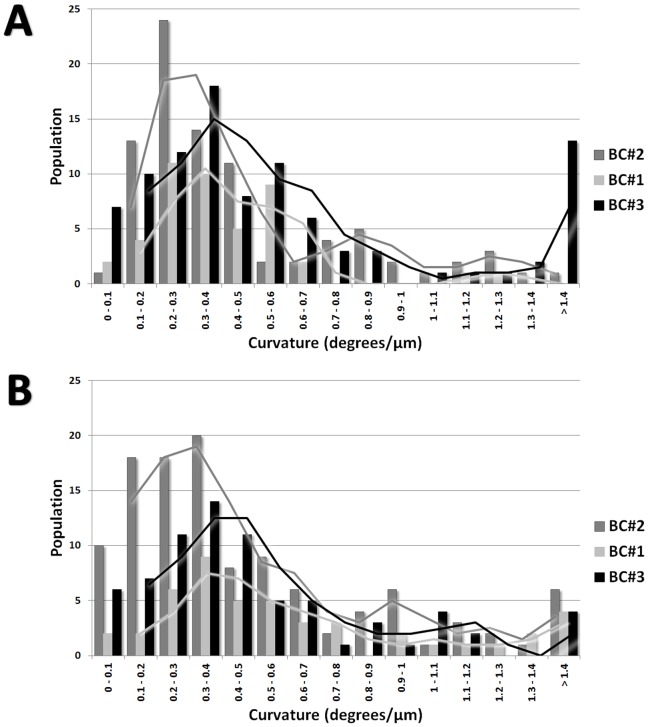
Distribution of branch curvature. (A) Comparison of all three biocontainers both for Flight and (B) Ground samples. Lines are the moving average of the data.

As for the hyphae curvature, the branch curvature did not show significant difference between flight and ground samples. The only difference to be noticed is the larger amount of branches with higher degree of curvature in the samples of BC#3 flight (Figure 10A, B). Here many hyphae did heavily fold, correlating well with the results presented in Figure 8.

**Figure 11:**
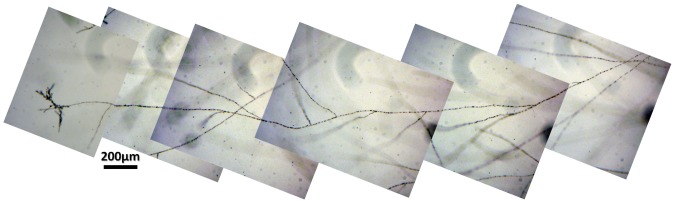
Long submerged hyphae ending with a microcolony.

Since in-flight the submerged growth was important, we also performed depth profiles measurements on all BC, except for the ground BC#3 in which the aerial mycelium reached the edge of the culture plate producing blurred and dark images. The flight experiment produced hyphae that extended in depth over long distances (Figure 11), in some cases very close to the edge of the culture plate and always initiating new colonies (Figure 6). This was not observed for the ground samples during the timeframe of this experiment.

### Microcolonies

Under spaceflight conditions, microcolonies evolved from a submerged hyphae extending towards the edge of the culture plates far away from the originating initial colony (Figure 6 and 11). Normally, individual hyphae exhibit an apical dominance; the growing tip suppresses branching in its vicinity, but sub-apical cells may generate new hyphae by lateral branching. In flight, some of the submerged hyphae lost the apical dominance and a chaotic branching took place. Microcolonies started in the depth of the substrate as a very dense mycelium, and then submerged hyphae oriented themselves towards the surface where oxygen is available, as proven by tests done on ground controls. Such microcolonies were observed only in the flight samples and their development was dependent on the age of the colony. We observed on pictures of BC#2 and BC#1 taken just after landing submerged mycelium looking like white dots. While, on the pictures of BC#3, the microcolonies had already reached the surface of the substrate and sporulated between FD9 and return; in BC#2 the microcolonies became visible on the surface of the substrate 3 days after return, in BC#1, 4 days after return. Similarly, microcolonies were found inside the substrate (white dots) in samples grown on the RPM (Random Positioning Machine) in the frame of ground studies (unpublished results) and in ground samples, which have been incubated for much longer time than the duration of the mission.

**Figure 12:**
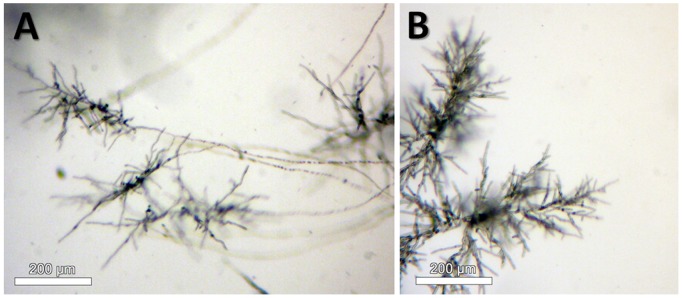
New colonies at their initial stage of growth. (A) Long submerged hyphae network leading to the microcolonies. Image taken at a depth of 100 µm. (B) Image taken from the outside of the microcapsule, no hyphae visible from this side.

**Figure 13:**
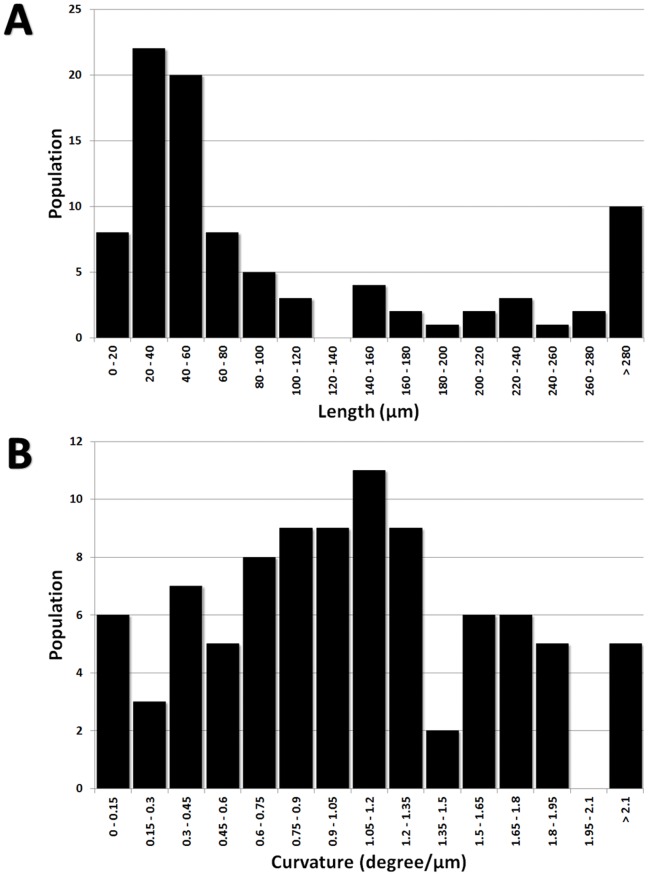
Microcolonies of *U. chartarum* developed in flight. (A) Distribution of hyphae length. (B) Distribution of curvature.

**Figure 14:**
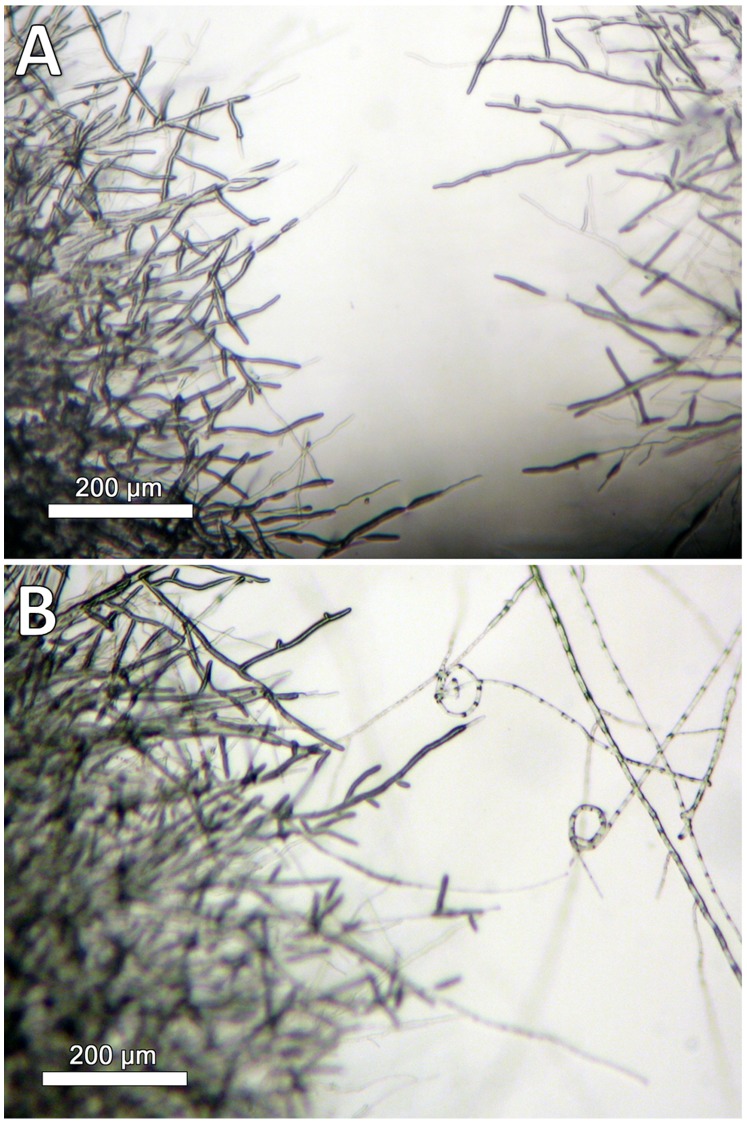
Microscopic images of the interface of microcolonies identified in BC#3 in flight experiment. (A) Interface between two new microcolonies. (B) Interface between the main and the new colony.

Microcolonies have the same characteristics as the initial colonies but are of smaller size (2–5 mm in diameter). They are constituted of aerial and submerged mycelium (Figure 12A, B). Hyphae of microcolonies are shorter and wider than those from submerged mycelium. In fact, the length of the microcolonies’ hyphae are mostly around 20–60 µm (Figure 13A) in comparison with the average of 200 µm in the main colonies (Figure 7). The degree of curvature (Figure 13B) is much larger in microcolonies (average about 1.1 degree/µm) than in the initial colony (0.1–0.12 degrees/µm). The limit between 2 microcolonies is made of healthy submerged hyphae (Figure 14A), differently between the initial and new colony folded and empty hyphae can be found (Figure 14B).

## Discussion

*Ulocladium chartarum* has been isolated from mural paintings where it develops fully viable aerial, sporulated mycelium on the surface of frescoes and submerged mycelium underneath it. It is a biodeteriogen of cultural heritage, with dark-brown spores and supposed to be very resistant to UV and low temperature like *Aspergillus terreus* [31] and possibly like *Cryomyces antarcticus* [28].

In the present study we demonstrate that *U. chartarum* colonies are able to grow under spaceflight conditions similarly to studies on other microorganisms [13], [32], [33]. The morphology differences observed among the flight, the ground, and the laboratory samples show that the environmental conditions are highly influencing the growth of the colonies. Actually, the environment of the closed biocontainer compared to the laboratory environment (only culture plates) has already an important effect, reducing the growth rate of the colonies. The major impact of the biocontainer’s environment observed on the aerial mycelium as well on spores and the growth of submerged mycelium is a strategy of the colonies to survive the stressors. The growth of aerial and submerged mycelium in parallel and the absence of microcolonies in the laboratory control is a proof of the absence of stressors. Spaceflight enhanced the rate of growth of the submerged mycelium and reduced the time of microcolonies development. Tests performed on ground showed that the oxygen inside the culture plates is not depleted during the duration of the experiment. This study shows that spaceflight is also one important factor having an influence on the morphology, the extended development of submerged mycelium, the enhancement of exudate drops formation, and the rate of growth. Thanks to the three different growth stages inside the three biocontainers, it could be shown that the longer the colony is grown in space conditions the bigger is the difference with the ground grown colony. As similar observations have been made on samples grown under simulated microgravity on the Random Positioning Machine (unpublished results) it suggests that microgravity is the driving factor and less the exposure to higher radiation or the high g-loads and vibrations experienced during launch and landing. This affirmation is also supported by the fact that, in space, repair of radiation-induced DNA damage in bacteria was close to normality [34]. Also Leys et al [35] reported similar results for *Cupriavidus metallidurans* strain CH34 in a flight experiment performed in the same type of biocontainer.

The rate of growth of *U. chartarum* colonies for the first five days was significantly higher in spaceflight conditions than on ground. This result shows that cell division and branching are not inhibited in space and cells proliferate faster. Different results have been found in case of *Saccharomyces cerevisiae*, which showed a lower growth in flight [13]. It is considered that differences are due to the experiment set up as well as to species specificity.

The effect of microgravity can be found at relative weight, circulation of heat and at cell level, also affecting the streaming of the cytoplasm [36]. For example, it was found that the velocity of cytoplasm streaming of *Physarum polycephalum* increased by 120% in microgravity [37]. Improved cytoplasmic streaming plays an important role in metabolism by fostering the transport, mixing and uptake of nutrients [38], [39]. Changes of the tension forces on the cytoskeleton and of the ion channel function will also finally influence the cell behaviour. The higher rate of growth of *U. chartarum* on solid substrate and the increased secondary metabolites elaborated on the surface of the colonies as exudates could be considered as an effect of reduced gravity on the organism itself or on processes taking place at the sub-cellular level like altered fluid uptake from the agar which is in accordance with results got with other fungal species like *Humicola fuscoatra* [39]. The higher growth rate as a response to microgravity is also a consequence of the fact that cell proliferation rate is higher than in gravity. In orbit similar results have been obtained at cellular level for other microorganisms like *Escherichia coli* [33], [40], [41], [42] or plants like *Arabidopsis thaliana* [43]. The growth stop observed after FD5 for aerial mycelium in-flight and ground was preceded by hyphal branching and loss of the dominance of the main hyphae, leading to apical branching, chaotic lateral branching, and vacuolization. Similar results were obtained with *Aspergillus nidulans* [44] and *Neurospora crassa* [45], [46].

The most prominent difference between flight and ground samples is the presence of long submerged, fully viable mycelium and the development of microcolonies at the edge of the culture plates in the flight samples. We saw that healthy hyphae orientated towards the depth of the substrate, by growing and branching in the horizontal and vertical position. The very long, almost unbranched hyphae grew rapidly towards the edge of the culture plates away from the main colony. It is well known that in areas rich in nutrients the mycelium branches and grows slowly maximizing the amount of nutrients it can extract; in contrast in areas low in nutrients the hyphae grow more rapidly and with little branching. Possible factors, like biosynthesis of toxic compounds, CO_2_ accumulation, and the quasi absence of air convection in space, could favour the growth of submerged mycelium having extreme apical dominance and finally the appearance of microcolonies, due to heavy random lateral branching, as a strategy to assure the survival of the colony and to find less harsh growing conditions. Exudates or liquid droplets were found on the surface of colonies in all experimental conditions but they were larger and numerous in flight than on ground. Coletelo [47] suggested that exudates are associated with actively growing mycelia and McPhee [48] that the process of exudation is of physiological significance and closely associated with colony aging. The exudation of liquid droplets is common for *Ulocladium chartarum* as well as for other fungal species like *Sclerotinia sclerotiorum* [48], [49]. A proteome-level study revealed that proteins present in the exudates could be classified into several functional categories including secondary metabolism [49]. Different secondary metabolites have been isolated from different species of *Ulocladium*: botralin (*Ulocladium botrytis*) [50], curvularins (*Ulocladium atrum*) [51], ulocladol A and ulocladol B (*Ulocladium chartarum)* [52]. Space conditions and the closed environment of the biocontainer could lead to an amplification of secondary metabolism, and excretion of metabolites, faster accumulation, and higher concentrations close to the colony, which as such act as stressors/toxins. We assume that environmental stress force the species to show its versatility to switch to develop exclusively submerged mycelium respectively invasive growth. There seems to be a network of sensing mechanisms and of signalling pathways able to transmit the information on aerial and nutritional status of the environment to cellular level directing the hyphal growth to the depth of substrate.

The viability of aerial mycelium was strongly affected by the experimental set-up (biocontainer), as in laboratory conditions the aerial mycelium was always fully viable. Our results are different than those obtained by Purevdorj-Gage et al [53] in low-shear modelled microgravity and by Walther et al [12] or Van Mulders et al [13] in real microgravity; these authors showed that viability of *Saccharomyces cerevisiae* was not affected by being cultured on liquid or in solid substrates. The unviable aerial mycelium is believed to be a consequence of the biosynthesis of secondary metabolites like toxins that accumulated extracellularly instead of cytoplasmatically, damaging the cellular structure and finally leading to the collapse. Fang et al [54] found similar results when growing *Escherichia coli* in different conditions showing that the accumulation of microcin B17 was shifted from cellular to extracellular site. Hyphal death in *U. chartarum* colonies can also be a consequence of autolysis and disorganization of internal cell constituents, loss of plasma membrane and cell wall integrity. We agree with the idea that during the life cycle of *U. chartarum* the mycelium undergoes a highly regulated process of programmed cell death [55] which is normal but the environmental conditions inside the biocontainers accelerate it. In stressed conditions the colony elaborates a new strategy to survive for a short time developing submerged mycelium and for a long time sporulating microcolonies on the surface of the substrate. By spreading the spores this species is able to survive, and preserve to extend throughout evolution.

Our results support the idea that space conditions have a positive effect on cell division and branching in submerged mycelium. In the depth of the substrate where invasive growth was identified, hyperbranching of submerged hyphae had been found in flight. It is possible that space conditions affect the regulation of Ca^2+^ influx at the tips, which are closely tied to hyperbranching. Kawano and Said [56] found that an excess of Ca^2+^ acts as an environmental suppressor of hyperbranching under several circumstances. Watters and all [57] found that a diverse gene network coordinates branching. Hyperbranching could also be an effect of spaceflight on disruption of genes involved in controlling branching. Mechanical stress induced by margins of the culture plates, microgravity itself and depletion of oxygen in the depth of substrate could induce a switch of very short hyphae from horizontal to vertical towards the surface of the substrate where new sporulated mycelium is developed.

A complex colony morphotype of *U. chartarum* is evident as a response to spaceflight conditions: in the same culture plate there is the initial old colony with irregular margin due to bended and folded hyphae, submerged mycelium and many microcolonies on the substrate developed by hyperbranching of submerged hyphae in the area with rich nutrient and no by-products. For *Saccharomyces cerevisiae* the concept “colony morphology response” was developed by Granek and Magwene [58] saying that complex colonies produce an extensive extracellular matrix that is absent in simple colonies and a protective role against a hostile environment has been proposed.

### Conclusions

This study clearly indicates that *Ulocladium chartarum* is able to grow under spaceflight conditions developing, as a response, a complex colony morphotype that has not been previously mentioned.

The colony growth cycle of *U. chartarum* provides a useful eukaryotic system for the study of fungal growth under spaceflight conditions. Here we prove that the growth of *U. chartarum* colonies is not affected by spaceflight.

In Spaceships and Space Stations *U. chartarum* and other fungal species could find a favourable environment to grow invasively unnoticed in the depth of surfaces containing very small amounts of substrate, posing a risk factor for biodegradation of structural components, as well as a direct threat for crew health.

Further investigations are needed to elucidate if the found complex colony morphotype is specific or general for other fungal species, as well as to explain the effect of spaceflight at cellular and physiological levels.
